# Tackling schistosomiasis in fisherfolk communities in Uganda: Enablers and challenges for implementing paediatric schistosomiasis mass drug administration from the perspective of district health authorities

**DOI:** 10.1007/s00103-025-04066-w

**Published:** 2025-06-06

**Authors:** Lisa Sophie Reigl, Isabelle L. Lange, Orkan Okan, Stella Neema, Andrea S. Winkler

**Affiliations:** 1 Department of Neurology, TUM University Hospital and Center for Global Health, School of Medicine and Health, Technical University of Munich, Munich, Germany; 2 Department of Infectious Disease Epidemiology and International Health, London School of Hygiene & Tropical Medicine, London, UK; 3 Health Literacy Unit, Department of Health and Sport Sciences, TUM School of Medicine and Health, Technical University of Munich, Munich, Germany; 4 WHO Collaborating Centre for Health Literacy, Department of Health and Sport Sciences, TUM School of Medicine and Health, Technical University of Munich, Munich, Germany; 5 Department of Sociology and Anthropology, Makerere University, Kampala, Uganda; 6 Department of Community Medicine and Global Health, Institute of Health and Society, University of Oslo, Oslo, Norway; 7 Department of Global Health and Social Medicine, Harvard Medical School, Cambridge, MA USA

**Keywords:** Schistosomiasis, Arpraziquantel, Preschool-aged children, District health authorities, Mass drug administration, Bilharziose, Arpraziquantel, Kinder im Vorschulalter, Bezirksgesundheitsbehörden, Flächendeckende Medikamentenverteilung

## Abstract

**Background:**

Schistosomiasis remains a significant public health issue in Uganda, particularly among fishing communities. This study, nested within a larger community-based study (ADOPT) on introducing a new child-friendly praziquantel formulation for schistosomiasis, arpraziquantel, explored enablers and challenges perceived by district health authorities for implementing a mass drug administration (MDA) programme with arpraziquantel.

**Methods:**

Respondents in this cross-sectional study were purposively selected in Bugiri and Hoima districts, Uganda. Key informant interviews were conducted in July 2022 and thematically analysed using the World Health Organization health system building blocks framework.

**Results:**

In general, arpraziquantel is expected to be widely accepted, though some may oppose it for religious or political reasons, belief in witchcraft as a cause of schistosomiasis, or fear of side effects. High awareness of disease burden and the acceptance of the existing schistosomiasis MDA for adults and school-aged children were identified as enablers for successful implementation. Key implementation challenges include limited resources for sustained biannual MDAs, adequate remuneration of village health teams (VHTs), inadequate staff training, time constraints, uneven VHT workload, and weaknesses in the referral systems. Migration, difficulties sensitising mobile parents, and delayed meals due to poverty—complicating drug absorption requiring a full stomach—further hinder efforts.

**Discussion:**

This study underscores the importance of addressing health systems’ challenges through targeted measures to ensure effective and sustainable MDA of arpraziquantel, including enhanced training of VHTs and other staff, sensitisation of parents and key stakeholders at all levels, and adequate responses to potentially emerging side effects.

**Supplementary Information:**

The online version of this article (10.1007/s00103-025-04066-w) contains supplementary material, which is available to authorized users.

## Abstract (de)

**Hintergrund:**

Bilharziose ist in Uganda nach wie vor ein bedeutendes Problem für die öffentliche Gesundheit, insbesondere in Fischergemeinden. Diese Studie, die in eine größere bevölkerungsbasierte Studie (ADOPT) zur Einführung einer neuen, kinderfreundlichen Arzneimittelformulierung gegen Bilharziose (Arpraziquantel) eingebettet war, untersuchte die fördernden und hinderlichen Faktoren, die von den Gesundheitsbehörden auf Bezirksebene bei der Umsetzung eines Programms zur flächendeckenden Verteilung von Arpraziquantel gesehen wurden.

**Methoden:**

Die Befragten dieser Querschnittsstudie wurden gezielt in den Distrikten Bugiri und Hoima, Uganda, ausgewählt. Die Interviews mit Schlüsselinformanten wurden im Juli 2022 durchgeführt und anhand des WHO-Framework zu Bausteinen des Gesundheitssystems thematisch ausgewertet.

**Ergebnisse:**

Es wird erwartet, dass Arpraziquantel weitgehend akzeptiert wird, auch wenn teilweise Ablehnung basierend auf religiösen oder politischen Gründen, dem Glauben an Hexerei als Ursache von Bilharziose oder Angst vor Nebenwirkungen erwartet wird. Ein hohes Bewusstsein für die Krankheitslast und die Akzeptanz der bereits bestehenden Bilharziose-Medikamentenverteilung für Erwachsene und Kinder im Schulalter wurden als Voraussetzungen für eine erfolgreiche Umsetzung ermittelt. Zu den wichtigsten Herausforderungen bei der Umsetzung gehören begrenzte Ressourcen für nachhaltige halbjährliche Medikamentenverteilungen, eine angemessene Entlohnung der Village Health Teams (VHTs), unzureichende Personalschulung, zeitliche Einschränkungen, ungleichmäßige Arbeitsbelastung der VHTs und Schwächen in den gesundheitlichen Überweisungssystemen. Migration, Schwierigkeiten bei der Sensibilisierung von Eltern und verspätete Mahlzeiten aufgrund von Armut – was die Absorption des Medikaments, das einen vollen Magen erfordert, erschwert – fordern die Umsetzung zusätzlich heraus.

**Diskussion:**

Diese Studie unterstreicht, wie wichtig es ist, die Herausforderungen der Gesundheitssysteme durch gezielte Maßnahmen zu bewältigen, um ein wirksames und nachhaltiges Programm zur flächendeckenden Verteilung von Arpraziquantel zu gewährleisten. Dazu gehören eine verbesserte Ausbildung von VHTs und anderem Personal, die Sensibilisierung von Eltern und anderen wichtigen Akteuren auf allen Ebenen sowie angemessene Reaktionen auf potenziell auftretende Nebenwirkungen.

## Background

Schistosomiasis is a waterborne neglected tropical disease (NTD) caused by parasitic trematode worms of the genus *Schistosoma*. In 2021, over 250 million people required preventive treatment, according to estimates by the World Health Organization (WHO) [1]. Although schistosomiasis can cause acute illness, it often leads to chronic signs and symptoms over time, with a global disease burden of 1.4 million disability-adjusted life-years in 2017 [2]. The disease is widespread in tropical and subtropical regions, particularly in communities lacking safe water and sanitation, with Africa accounting for at least 90% of individuals requiring treatment [1]. Children are among the most vulnerable groups.

A meta-analysis of data from 2003 to 2020, stratified by region, reported that preschool-aged children (PSAC; ≤ 5 years) in East Africa had a pooled prevalence of schistosomiasis of 27% (95% CI: 12–43) [3]. In Uganda, this trend is reflected by a 36.1% prevalence among children aged 2 to 4 years, higher than the national prevalence of 25.6% [4]. Despite this, treatment campaigns have primarily focused on school-aged children and, in some regions, adults.

In Uganda, as in most countries, mass drug administration (MDA) excludes PSAC [5] because praziquantel, the primary medication, is officially licensed only for children aged 4 years and older [6]. However, growing evidence has debunked assumptions that young children have minimal exposure to parasites and infection [7–11]. In response, the WHO updated its treatment guidelines in 2010 to include preventive chemotherapy for children older than 2 years [12–14]. To close the treatment gap for younger children, the Pediatric Praziquantel Consortium (PPC) developed arpraziquantel, a child-friendly praziquantel formulation. The PPC’s ADOPT^1^ team, which is conducting a corresponding implementation research programme, leads its pilot distribution in Côte d’Ivoire, Kenya, and Uganda [15], with the first child treated in March 2025 [16].

Sustaining high coverage of MDA programmes frequently faces several challenges, including limited resources, local opposition, low compliance, and waning political and community support [17, 18]. Addressing these barriers requires strengthening governance at the district level, which lies at the intersection between national-level plans and community realities, where health officials play a pivotal role in planning, executing, and overseeing MDA campaigns [19]. District health leaders are in the position to offer critical insights into local health behaviours, people’s health literacy, gaps in health education and outreach, and resource limitations [20]. They are responsible for essential functions such as managing medication supplies, training health workers, and responding to health system disruptions [21]. Their involvement is vital for tailoring NTD programmes to local realities, increasing programme acceptance and impact [22].

The WHO emphasises that decentralised, district-led approaches to NTD control are more effective because they strengthen local capacity and enable interventions to adapt to community needs [12]. This localised approach is a core strategy for achieving the 2030 NTD elimination targets, as it ensures the sustainability and integration of initiatives such as paediatric MDA programmes into broader health system efforts, fostering greater community trust and programme adherence [23].

As part of this effort, a social science–driven implementation research study was conducted to inform the design and delivery of arpraziquantel to PSAC [24–26], alongside advocacy and social mobilisation activities across seven districts. This study forms part of the larger mixed method ADOPT study, spanning three countries and various stakeholder levels, aiming to identify enablers and challenges perceived by district health authorities in Uganda in advance of implementing a new paediatric MDA programme for schistosomiasis.

## Methods

### Setting

The study was conducted in two districts: Bugiri, close to Lake Victoria, and Hoima, at Lake Albert. The sites are mostly rural, with some areas described as hard to reach, accessible only by boat and motorbike.

The prevalence of schistosomiasis in these regions is above 30%, which can be traced back to their proximity to the lakes and insufficient access to safe water and sanitation. An annual MDA with praziquantel is conducted at schools for schoolchildren and in the community for children above 5 years of age who do not attend school, as well as adults. In this case, praziquantel is distributed through a door-to-door approach by village health teams (VHTs), which are equivalent to community health workers in other countries, or at fixed points, which can include the nearest health centre or a designated community location, facilitated by VHTs or health professionals.

The study locations are characterised by poverty, with many houses being makeshift and few having their own latrine. Safe water sources are scarce, and only a few villages are connected to the electricity grid. The effects of the COVID-19 epidemic (2020–2022), a fuel crisis, and continuous inflation have reached an unmanageable level, compounding challenges alongside high unemployment rates.

### Study design and sampling

Nested in a more extensive cross-sectional mixed-methods observational study, this analysis used data from the qualitative arm of the selected respondent group. We conducted in-depth interviews with six health authorities in two districts, including a district health officer, two district vector control officers, a district surveillance focal person, a district principal health inspector, and a district health educator (five men and one woman, DA1–DA6). The sampling followed a purposive selection of respondents.

### Data collection

Researchers from Makerere University and the Technical University of Munich Hospital conducted individual audio-recorded in-depth interviews in July 2022. The interviews lasted, on average, 1 h 28 min, ranging from 51 min to 2 h 26 min. Ethical approval was obtained from the Ethics Committee at the Technical University of Munich (731/21 S), the Makerere University School of Social Sciences Research Ethics Committee (MAKSSREC 01.22.528), and the Uganda National Council for Science and Technology (SS1269ES). Participants voluntarily joined after providing written informed consent, following receipt of an information sheet, verbal explanations, and opportunity to ask questions.

### Data analysis

After transcription, interviews were thematically analysed using the WHO health system building blocks framework. This framework outlines six core components determined integral for strengthening health systems: leadership and governance; health service delivery; health system financing; health workforce; access to essential medicines (also named medical products, vaccines, and technologies); and health information systems [27, 28]. The analysis is outlined in the following results section. Additional quotes organised by building block are available in the supplementary material.

## Results

The results are structured along the WHO health systems building blocks, though the boundaries between these categories and themes and topics overlap. A further theme concerning parental acceptance was added to highlight the importance of community attitudes in health campaigns.

### Leadership and governance

Schistosomiasis control requires multisectoral programme efforts, and district health authorities clearly articulated that collaboration with other sectors and politicians is an important part of their work. Regular meetings, such as weekly technical meetings, facilitate information flow. For the introduction of the arpraziquantel MDA, collaborative meetings with education sector stakeholders, politicians, and VHTs, along with sensitisation meetings for parents, are envisioned.

District authorities will continue their established roles within the existing MDA framework, while local political leaders, as well as religious and traditional leaders, will remain pivotal in mobilising communities for programme acceptance. However, collaboration with communities faces challenges, such as the unavailability of key personnel due to competing health and educational activities, as noted by two respondents from Hoima.

### Health service delivery

A major facilitator for implementing the arpraziquantel MDA is the long-established infrastructure for the schistosomiasis MDA targeting school-aged children and adults, which stakeholders and communities are already accustomed to. District health authorities expected that arpraziquantel would be well received if community sensitisation was conducted to address concerns about potential side effects. Our informants reported previously receiving training for the existing MDA targeting schoolchildren and adults. They envision that similar training would be organised, allowing them to actively participate in introducing the arpraziquantel MDA.

Health campaigns for treating schistosomiasis in school-aged children have been largely successful, according to a respondent from Hoima, citing the absence of community opposition and the support of cooperative teachers who facilitated the process through preregistration of children and height measurement. However, informants identified challenges such as insufficient time for MDA preparation and limited mobilisation of parents, which affected their overall participation. The MDA planning involves VHTs because of their knowledge of their communities, their role in providing information about health campaigns (sensitisation and mobilisation), and, in some cases, their responsibility for the distribution of drugs. However, the geographical size of local councils can lead to some VHTs overseeing larger populations, complicating service delivery and timely MDA.

Furthermore, delayed release of funds, lack of transportation, and insufficient resources for training and advocacy hinder the effectiveness of current programmes, particularly at the community level. Generally, transporting goods and people is difficult in these districts. The road network poses a challenge for distributing drugs, especially during rains when some roads, especially in the “hard-to-reach” areas, are impassable. One informant acknowledged that her team was not adequately prepared for a health campaign, as her other responsibilities and the limited drug supplies caused delays, resulting in her subcounty being the last to complete the MDA.

Challenges in sensitising transient and mobile parents hinder the effectiveness of health campaigns in both districts. A respondent from Bugiri expressed concern that treatment consistency would be disrupted by the absence of fishers and their rapid migration patterns. Meanwhile, a respondent from Hoima identified the lack of continuous, targeted health promotion messages about schistosomiasis reaching mobile populations as a significant barrier to programme success.

Drug distribution could use a door-to-door approach or occur at fixed points such as health facilities or village meeting spots, suggested a Hoima respondent. Fixed-point distribution requires strong community mobilisation, advocacy, and meticulous time management but poses challenges, such as ensuring that children eat beforehand and managing noise and other disruptions. However, this approach has the potential to be integrated with existing child health campaigns, such as child health days. Door-to-door distribution, preferred by some, provides better access to children under 5 years and privacy for drug recipients. A Hoima respondent noted that MDAs are timed to take place after lunch since most community members skip breakfast, and the drugs perform better on a full stomach. A Bugiri informant stressed the importance of directly observed treatment to ensure that patients take the medication.

An organised response plan for managing drug side effects is essential, as fears about the drug’s safety or suspicions of malicious intent can exacerbate community distrust.

“People tell a lot of lies about the drug: ‘It is bad’, ‘It is dangerous’, ‘It kills’, ‘The whites want to kill us’, etc.” (Bugiri, DA3)

Adverse reactions such as nausea, diarrhoea, and headaches, often occurring shortly after intake, amplify these fears. Without follow-up by VHTs or health professionals, signs and symptoms are misattributed to the drug rather than to the body’s response to dying worms, especially in individuals with high worm loads. Well-informed and well-prepared teams should promptly address reactions during and up to 1 week after MDA, with guidance and support from the National Drug Authority. Minor cases can be managed at lower health centres, though access to medication such as antihistamines is often limited. Therefore, comprehensive follow-up is essential to reassure patients and to supervise adequate management of side effects.

### Health system financing

Respondents’ perspectives underscore the significant challenges already faced in funding health programmes at the district level. Limited financial resources hinder the procurement of drugs, treatment of individuals, and support for VHTs and technical staff involved in activities such as health education and drug distribution. Programmes such as NTD control have faced delays due to funding constraints, reducing the frequency of treatments.

“If you don’t have financial support, then these people will not be treated.” (Bugiri, DA2)

Historically, the availability of drugs against schistosomiasis for MDAs in Uganda has depended on donors. One respondent from Hoima suggested that treatment should be carried out twice per year to reduce prevalence more effectively, but insufficient funding is an obstacle.

In the past, payments to VHTs in Hoima had been delayed due to bureaucratic procedures. However, a new system was implemented, allowing funds to bypass the district level and be disbursed directly to subcounty personnel.

### Health workforce

The VHTs are known to be knowledgeable about their community, e.g. they know its families and the number and ages of children. Informants noted that VHTs receive small, delayed payments, often complicated by mobile money system issues such as errors and bounced transactions. They stressed the need for timely payment, clearer communication about payment methods, and better funding to prevent VHTs from losing motivation, as inadequate compensation has led to poor participation by VHTs in community-based activities such as COVID-19 vaccination campaigns. While VHTs are generally trusted within their communities, one respondent noted they might not always be fully respected or viewed as credible sources of information compared to trained health workers. This perception could undermine their effectiveness in promoting health initiatives.

When asked what needs to be done to ensure that health workers and VHTs can effectively perform their assigned tasks, respondents highlighted the importance of both training (“empowering them through knowledge”, DA4) and providing (monetary) facilitation, along with feedback on their performance. Most informants emphasised the need for more appropriate VHT training. Training sessions are often limited to a single day, restricting practical learning on disease transmission, control strategies, and data management.

“I know the training pieces are sometimes inadequate, depending on who’s training them, because we give them all the materials in one day, whereas I’d prefer we give them the doses [=drugs], and let them practice.” (Hoima, DA4)

Beyond the crucial role of VHTs, health professionals are essential to ensuring the effectiveness of health interventions. Health workers are particularly critical in managing the drug’s side effects, requiring active monitoring and accurate documentation of reactions through pharmacovigilance forms. However, insufficient funding for wages and recruitment plus poor supervision hinder their ability to perform these essential tasks, as noted by another informant.

### Access to essential medicines

Most community members appreciate the free provision of preventive chemotherapy for school-aged children, made possible by donor contributions. In Bugiri, district authorities reported that parents had expressed a strong desire for a suitable treatment for younger children, and district authorities articulated concerns about the risks of unregulated medication use. However, the provision of free drugs for acute treatment beyond MDAs is not guaranteed:

“At the management level, the drug is not always there, and if it is there, it is very expensive. If you tell someone to go and buy from the pharmacy, they cannot afford it.” (Bugiri, DA3)

Ensuring an uninterrupted and free drug supply could significantly reduce disease transmission.

### Health information systems

A respondent from Hoima reported that schistosomiasis is underdiagnosed, as treatment focuses on signs and symptoms rather than underlying causes, leaving cases unregistered. Similarly, deaths are often misattributed to malaria due to shared signs and symptoms such as anaemia.

“[…] By missing such important information, we are not informing, the system is not informing the ministry that bilharzia is probably a very big problem.” (Hoima, DA6)

Building on this, another respondent highlighted the challenges of misdiagnosis, noting that schistosomiasis is sometimes confused with diseases like Ebola due to limited training among health workers. These factors contribute to the underreporting of schistosomiasis prevalence and mortality.

Other systemic issues exacerbate these challenges. A Hoima district officer reported praziquantel MDA coverage rates above 80% in most areas but questioned the data’s reliability due to challenges in capturing treatment numbers across schools and communities. Record-keeping issues, such as discrepancies in medication distribution records, further undermined accuracy. In Bugiri, campaigns faced a lack of public address systems and inadequate data management, compounded by an only partially functional health information system.

Pharmacovigilance efforts face their own set of obstacles. In Hoima, VHTs were described as alert and proactive in reporting suspected illnesses from the community to higher-level authorities. However, these efforts are often undermined by a referral system that lacks robust follow-up and feedback mechanisms. This communication gap between different levels of healthcare providers significantly hampers effective pharmacovigilance and the monitoring of adverse events.

### Parental acceptance of the new health programme

We added parental acceptance as a key theme to account for factors influencing parental acceptance, which are not part of the WHO health system building blocks. Respondents generally expected parents to welcome the new treatment, though one respondent stressed that acceptance should not be taken for granted. Concerns arose from past incidents in which mass treatments or food distributions allegedly were linked to child deaths or illnesses, creating scepticism or concerns about new health programmes. One informant shared:

“I believe the acceptance level will be very high. […] I do not anticipate much except for when we introduce it, and there happens to be severe side effects because that one can create stigma and lead to some caretakers shying away.” (Hoima, DA6)

Some people attribute schistosomiasis to witchcraft and initially consult witch doctors, delaying proper treatment (these beliefs exist in both districts but are more widespread in Bugiri). Such beliefs can lead even local leaders to privately doubt health campaigns while offering public support.

Furthermore, MDAs can become politicised, and political opposition can influence the uptake of health programmes. Respondents noted that some individuals reject initiatives simply because they are associated with the government, regardless of the programme’s benefits. This was highlighted in Bugiri, where certain religious sects rejected medical treatment and government programmes entirely. As one informant from Bugiri explained:

“We have a sect called Njiri Nkaru (dry gospel). These people don’t believe in treatment. They don’t want to hear or support any government programmes. They don’t want, say, even to swallow tablets. This sect, they are already protected by the powers of the lord.” (Bugiri, DA2)

Migration was another factor affecting parental acceptance. Migrants from Congo were cited as having different attitudes and were expected to be more resistant to the new treatment. Competing priorities also influence participation, as parents balancing daily income-generating activities may be unable to prioritise MDA participation. Despite these challenges, community acceptance can increase over time. Respondents noted that some community members might prefer to observe the effects on those who initially receive the treatment before deciding to participate, as seen with the COVID-19 vaccinations.

To address these challenges, respondents recommend effectively informing parents through ongoing community education and awareness efforts about the programme’s importance as well as schistosomiasis prevention and treatment. Health educators should be well-informed and deliver clear, evidence-based messages about the drug’s safety and efficacy, highlighting successful implementation elsewhere. Besides inception meetings at all levels, it was suggested that information be distributed through posters, pictures showing cured patients, drama plays, health talks, and the radio. At present, appropriate information, education, and communication materials for schistosomiasis are not being distributed.

## Discussion

Interviews with district health authorities revealed both enabling factors and challenges for implementing an arpraziquantel MDA in Uganda. These challenges span all components of the WHO health system building blocks, highlighting the complexity of integrating new health programmes.

Applying the WHO’s health systems thinking approach helps identify leverage points within health systems to design, implement, and evaluate more effective public health interventions [28, 29]. However, our experience revealed limitations in the rigid segmentation of health system components, as it overlooks their interconnections. Other researchers have raised similar concerns, arguing that focusing on discrete building blocks impedes a holistic understanding of health systems dynamics [30]. Given that the building blocks do not fully address the demand-side factors influencing community behaviour [28, 30], we included parental acceptance of the new health programme as a key theme in our analysis.

A key enabler for an arpraziquantel MDA is the existing infrastructure for schistosomiasis MDA and the community’s familiarity with MDA procedures. Regarding parental acceptance of the new health programme, district health authorities generally anticipate a positive reception of the new medication if community sensitisation effectively addresses possible side effects. However, it was also highlighted that community acceptance could not be taken for granted: Concerns persist due to past incidents, the divergence between biomedical and local understandings about the causes of schistosomiasis, and hesitation of some groups (e.g. migrants) due to political, religious, and cultural factors or fears about the drug’s safety or suspicions of malicious intent. To address these, respondents emphasise the need for multilevel stakeholder sensitisation, including community, religious, and traditional leaders; continuous community education; clear communication (based on health literacy principles) about the programme’s safety and benefits; and the provision of well-crafted, pictorial informational materials to build trust and address scepticism. To ensure equitable programme coverage, it is essential to develop targeted engagement strategies for critical subpopulations. In addition to VHTs and parents, the involvement of other trusted community actors—such as leaders of fishermen’s associations and women’s groups—was identified in interviews with other informants (such as parents and community members) as essential for reaching and engaging critical population groups [24]. Specific concerns and misconceptions can be addressed by using culturally and linguistically appropriate communication.

For health service delivery and workforce preparedness, respondents highlighted the need for comprehensive training to prepare all stakeholders for the new programme. However, they criticised the existing training for its poor quality, insufficient duration, and omission of practical exercises. Additionally, appropriate training materials are currently not being distributed. For the future, respondents recommended extending the duration of the training and incorporating more practical exercises and informational materials to enhance understanding and retention. In this context, workforce development and training should include professional health literacy capacity building for the health professional [31–33] to better respond to community health (literacy) needs and demands. Considering that health campaigns at schools seemed effective but health promotion messaging revealed shortcomings, the health literacy lens could be applied to user-centred health communication, while health campaigns at schools might be adapted to systematically link health literacy and schistosomiasis education.

The VHTs play a role in referring suspected schistosomiasis cases and individuals experiencing side effects after MDA. However, the referral system’s effectiveness is hindered by weaknesses such as inadequate follow-up and feedback. Implementing electronic tools could strengthen the referral process by providing VHTs with timely updates on patient outcomes, fostering trust within communities and enhancing patient support. Well-prepared teams should promptly address adverse reactions during and up to 1 week after MDA. In rare cases, and particularly in areas co-endemic with *Taenia solium*, neurological side effects may occur in individuals with clinically silent neurocysticercosis [34, 35]. Data on neurocysticercosis in young children in MDA areas remain scarce, which is why children with a history of nonfebrile epileptic seizures—used as a clinical proxy for neurocysticercosis—are often excluded from MDA a priori.

Another challenge is the availability of drugs. In our research sites, drugs were often not available at health facilities for treatment of acute cases, and drug supplies for MDAs were regularly delayed. While Merck has committed to providing 250 million tablets of praziquantel per year until elimination [36], their delivery needs to progress through a complex and costly supply chain until they reach the remote communities most at risk for NTDs [37]. One respondent favoured biannual treatment for high-prevalence regions instead of annual treatment, an approach supported by the literature [38]; however, this approach is hindered by the lack of resources.

The same informant hinted at the possibility that decision-makers may underestimate the true burden of schistosomiasis, which could be due to unreliable data on its prevalence and mortality. Schistosomiasis-related signs/symptoms are often attributed to other diseases, such as malaria or even Ebola disease, further obscuring the scale of the problem. In addition, death registries are not always reliable.

Looking further at surveillance and monitoring, some district authorities view data collected by VHTs on MDA coverage as unreliable. Similarly, other studies have highlighted discrepancies and incomplete data in MDA registers, with surveys revealing gaps between reported and actual treatment coverage. These inconsistencies can significantly affect future logistics and drug planning [39, 40]. Community drug distributors’ literacy levels are reported to contribute to the data quality [22]. Although improving their literacy levels may prove challenging in the short term, leveraging electronic data collection tools and systems offers a potential solution to the identified shortcoming. The successful utilisation of electronic tools for data collection and rapid transfer was demonstrated by a study on SMS-based reporting of NTD cases in Ghana and Malawi [22, 41]. Despite challenges such as the provision of smartphones to VHTs, the ability to ensure adequate training on digital literacy, difficulties in keeping devices charged in areas with limited electricity infrastructure, and unstable or absent network connectivity, electronic data collection tools have the potential to enhance decision-making processes and attract national funding. [22]. Ideally, such tools should be integrated into the National Health Information System to ensure sustainability and alignment with broader health system goals.

To achieve the long-term goal of elimination, WHO’s mapping recommendations, which classify the prevalence of an entire district based on the average prevalence from five schools, may be insufficient. More detailed prevalence data are needed to accurately identify and target hotspots for treatment [42–44]. Strengthening disease surveillance through improved data collection, enhanced diagnostics, reliable death registries, and awareness campaigns could help reveal the true burden of schistosomiasis, encouraging greater political commitment.

Leadership and governance are crucial for programme sustainability. Despite low health sector funding [45], respondents emphasised the need for decision-makers to prioritise NTD programmes. Evidence suggests that investment in NTD control yields high returns, with improved health outcomes contributing to national economic development [46, 47]. Furthermore, NTD control deserves greater attention from policymakers in low- and middle-income countries as an integral component of poverty reduction strategies [22] and its impact on other health measures. For example, *Schistosoma haematobium *has been linked to higher rates of HIV [48] and human papillomavirus [49] infections, particularly among girls. Given the instability in international NTD funding [50], there is an urgent need for local ownership, sustained political will, and domestic financial commitment.

For sustainable success, integrating NTD programmes into government planning and investments [51] and shifting from control to elimination strategies are pivotal. The latter requires intensified interventions; expanded treatment coverage; snail control; improved water, sanitation, and hygiene; enhanced health education; and stronger monitoring, evaluation, and surveillance systems [38]. The efforts should not only address the burden of disease but also monitor potential side effects after treatment, whether in health centres or through MDA campaigns.

## Limitations

This analysis is based on a limited sample of six interviews; however, findings were corroborated through triangulation with responses from other stakeholders in the broader study, which included participants at both national and community levels. Nonetheless, five of the six informants were male, which may have introduced a male-centric bias into the analysis. The presence of German researchers and their institutional affiliation with a consortium involving the Ministry of Health may have influenced participants’ responses, potentially prompting socially desirable answers or reluctance to express dissenting views. Although German researchers, supported by an experienced Ugandan team, interviewed only informants who were comfortable speaking English and were familiar with interactions involving foreigners while discussing their professional lives, the involvement of international researchers may have introduced implicit power dynamics. These dynamics—shaped by differences in nationality, perceived authority, and institutional affiliation—could have influenced the interview context and affected how participants framed their responses. We acknowledge that such positionalities may have impacted the openness of participants and recognise the need for ongoing reflexivity in international, collaborative research settings.

## Conclusion

This study highlights the insights that district health authorities have on the key enablers and challenges for implementing an arpraziquantel MDA for PSAC in Uganda. The existing MDA infrastructure and community familiarity with treatment processes offer a strong foundation for upcoming activities. However, challenges span all WHO health system building blocks, including health service delivery, workforce training, drug availability, financing, health information systems, and leadership. Addressing these challenges requires targeted measures such as robust and well-functioning health systems, including adequate and foremost timely pharmacovigilance; improved training for stakeholders at all levels; regular cross-sector inception and planning meetings; appropriate information materials; electronic data collection, including for the management of potentially ensuing side effects; and increased and sustained political commitment. To guide future scale-up and ensure systematic consideration of sustainability, acceptability, and fidelity, the use of a formal implementation science framework such as the Consolidated Framework for Implementation Research (CFIR) could be beneficial. Applying selected CFIR constructs in future phases would provide a structured approach to assessing contextual factors and implementation processes. This would facilitate comparison of results across contexts over time, while also supporting the adaptation and integration of arpraziquantel distribution to PSAC within national systems.

## Supplementary Information

Table 1: Quotes organised by the WHO building blocks

## Footnotes

1 Adoption of levo-praziquantel 150 mg for schistosomiasis by endemic countries. The name “levo-praziquantel” was later changed to “arpraziquantel”.

## Declarations

Ethical approval was obtained from the Ethics Committee at the Technical University of Munich (731/21 S), the Makerere University School of Social Sciences Research Ethics Committee (MAKSSREC 01.22.528), and the Uganda National Council for Science and Technology (SS1269ES). Participants voluntarily joined after providing written informed consent, following receipt of an information sheet, verbal explanations, and an opportunity to ask questions.
