# Supplementary material for: Tackling schistosomiasis in fisherfolk communities in Uganda: Enablers and challenges for implementing paediatric schistosomiasis mass drug administration from the perspective of district health authorities
Source: Bundesgesundheitsblatt Gesundheitsforschung Gesundheitsschutz. 2025 Jun 6;68(7):778–86. doi: 10.1007/s00103-025-04066-w (PMC12254165; doi:10.1007/s00103-025-04066-w)
Supplement: Supplementary file 1 — Table 1: Quotes organised by the WHO building blocks [file 103_2025_4066_MOESM1_ESM.pdf]

Supplementary material

Table 1: Quotes organised by the WHO building blocks

Theme	Sub-topics	Selected quotes
Leadership and governance	Stakeholders	The politicians are the key; if you want to succeed, you have to start with the local leaders. Especially our councillors at the district, the sub-county, then you go to the local council one, they are the governors of the real villages – like the man we were moving with when we first went to [name of the village], […]. Then also those cultural leaders, we can bring them on board, we can bring the herbalists on board, schools, teachers, religious sectors, etc. (DA2)
		The politicians, religious leaders, cultural leaders, businessmen and women. Traditional healers should also get involved. (DA1)
		[…] It is the education sector, the community sector, the political wing, and then some of the VHTs. (DA6)
	Roles of District Authorities	I will be part of the social mobilisation and sensitisation, I will be part of the advocacy team, I will be part of the training of trainers, I will do monitoring, which should give feedback meetings, that there is also a big gap when we treat, we get our data and we take, we do not go back to tell them how we performed, we don't go back to look at them, to ask them how they are feeling, that relationship also we should put it on board. (DA4)
		My involvement will be in mobilisation and health education. Training or skilling the lower cadres at the sub-county level downwards. And even support supervision during the implementation. (DA3)
		So, planning and supervision, follow-up, collecting data, and managing data, those are my roles; and mobilising communities. (DA1)
	Competing activities	[…] I was supervising that activity with my assistant. My assistant is the health in charge of the area, and in most cases, he was there on the ground, and I kept in touch, but I would not go there because of these other responsibilities which I had and actually, our sub-county was the last to finish the exercise because we received less drugs than we wanted because me as the health supervisor in the district I was engaged in some other things but later we had to finish. (DA5)
		In collaboration, the challenge is competing activities. You may need an inspector of school to be on your board, but you find them very busy. (DA4)
	Unequal distribution of VHTs' workload	Then also you find some VHTs, depending on the geographical demarcation of our areas, you find what we define as an LC [Local Council] is big; it has a population of over 300 people, and that is one, of course, by definition, in terms of when we are determining coverage and training, we train these people based on the

		communities which are gazette within the district. Then may be the one is finance, financial support. If you don't have financial support, then these people will not be treated. Like our district is a local district, we have no funds, enough funds. They can't run such a program, which can cost a lot of money. Procuring drugs, giving people some support t like the VHTs, even the technical staff to go down to health educate, then give out the drugs, etc. You may find out that the local district cannot do it if you people don't come in to help with arrangements. So, you find other VHTs handle a bigger population, which becomes a bigger challenge, and we, as we are saying, we think they should do the MDA within the shortest possible time, which is sometimes very challenging. (DA6)
Health service delivery	MDA infrastructure	They have been doing it [MDAs] once every year. For the last, I think, over ten years in order to reduce [schistosomiasis]. So, every year they do, the prevalence has been reducing. Every year we do until we are able to achieve less than five per cent, and now we are managing it as endemic, yeah. Those are manageable levels. The other time it was quite high. (DA1)
		Now, about those mass campaigns, first, they train the district supervisors who go and train the lower cadres at the sub-county. They are supposed to supervise the VHTs and the local leaders (LCs) because each one has roles and responsibilities to play. (DA3)
		No, it is the same, I will be part of the social mobilisation and sensitisation, I will be part of the advocacy team, I will be part of the training of trainers, I will do monitoring, which should give feedback meetings, that there is also a big gap when we treat, we get our data, and we take, we do not go back to tell them how we performed, we don't go back to look at them, to ask them how they are feeling, that relationship also we should put it on board. (DA4)
	Insufficient time for MDA preparation	In terms of time and preparation for the exercise, I saw a lot of speed, we did not give it enough time, because even in those advocacy meetings, the turn-ups were not very good, and it is hard for the community to mobilise because the community was easy to mobilise because the children to benefit were at school. But remember, these children belong to some parents, so parents needed to be well-versed and actually, I was talking to name of[the vector control officer] and I was saying that yes, now our target population is in the school, yes but I am looking at the parents as well, we have not had time to mobilise the parents but we didn't get resistance. (DA5)
	Unequal distribution of VHTs' workload	Then also you find some VHTs, depending on the geographical demarcation of our areas, you find what we define as an LC [Local Council] is big; it has a population of over 300 people, and that is one, of course, by definition, in terms of when we are determining coverage and training, we train these people based on the communities which are gazette within the district. Then may be the one is finance, financial support. If you don't have financial support, then these people will not be treated. Like our district is a local district, we have no funds, enough funds. They can't run such a program, which can cost a lot of money. Procuring

		drugs, giving people some support t like the VHTs, even the technical staff to go down to health educate, then give out the drugs, etc. You may find out that the local district cannot do it if you people don't come in to help with arrangements. So, you find other VHTs handle a bigger population, which becomes a bigger challenge, and we, as we are saying, we think they should do the MDA within the shortest possible time, which is sometimes very challenging. (DA6)
	Delayed fund releases, lack of transportation, and insufficient resources for training and advocacy	In collaboration, the challenge is competing activities. You may need an inspector of school to be on your board, but you find them very busy. Then, the medicine distribution, the challenge is in terms of funding fuel. (DA4)
		Then the roads deep in the community are also not good. The road network is another issue because the roads are not passable (DA3).
	Competing activities	[...] I was supervising that activity with my assistant. My assistant is the health in charge of the area, and in most cases, he was there on the ground, and I kept in touch, but I would not go there because of these other responsibilities which I had and actually, our sub-county was the last to finish the exercise because we received less drugs than we wanted, because me as the health supervisor in the district, I was engaged in some other things, but later we had to finish. (DA5)
	Reaching fisher families	You find that this time they have got treatment here in [name of bigger village], but next year when giving them another round, they [families] would have immigrated or shifted to another, so you find that they move according to how much they are fishing. If it's depleted in [name of the bigger village], tomorrow they go to another place where fish is being caught in big numbers.<br>Then we also have migration. There also these inlands [residents of inland areas], they come and settle at the lakeside there, but sometimes again they also move away, they may go like in [name of the bigger village] one time we had people who moved to [name of other district] that there is fertile land there. So, they had to go, and then we had a depletion of people in [name of a bigger village]. So, these immigrations also cause no consistent treatment. (DA2)
		[...] misconceptions remain at an individual level except in some communities where we have constant immigration, people come in from Congo, those who are not informed, they tend to have different beliefs that they have been bewitched, but this one I think one of the challenges is that much as we have health promotion, there is no continuous health promotion messages targeting specifically bilharzia. (DA6)
	Drug distribution	**Respondent:** You know the home is more comfortable and private to our parents and even to the children. Because let me tell you one very sensitive thing: Here in this place, parents do not count their children, and they associate it with dying. So, if you gather them in one central place and have children below 5

		years, 4 of them, already in the way of getting them, already, I am in the wrong, you stigmatise them because you cannot have those 4 children below five years, but some people have them, so it is a shame. I cannot come out with these children in the community, but if you find me at home, nobody will know my number of children.<br>[…] That's what they would do, and that's what will happen when children are merged in one place, a lot of noise because when they want to cry, you can't stop them from crying, they are hungry and that one will put an expenditure on the parents because they have to pack food, they have to pack mandazis when this child sees the other one is taking soda, they want it also.<br>**Interviewer: Okay, so we need to also facilitate them.**<br>**Respondent:** But if they are in a home environment, they would be eating whatever at home while you are busy doing the treatment. (DA5)
		Otherwise, since it is a very big requirement during MDA, it is a pre-requisite that MDA can always begin after lunch because that's when people because most of our communities do not receive what we call breakfast, they simply wake up and go to gardens and go to their places, so if you are saying they should have eaten by the time you are getting the drug, then they are telling you that by the time they are beginning to eat at 1, that is the time when you should begin your treatment, so if you are moving house to house, it becomes a challenge. (DA6)
	Organised response to side effects	You see community level has a problem of misconception. There is a lot of propaganda, especially to the school-going age children, and that may mislead the population. People tell a lot of lies about the drug: 'It is bad', 'It is dangerous', 'it is kills', 'The whites want to kill us', etc.<br>Then there is drug reaction. It is a very bad thing in the community. It must be handled immediately.<br>[…] Of course, with every mass campaign you must be prepared for the drug reaction. There is a team for handling those reactions. Even the National Drug Authority has that provision for drug reactions. Any adverse drug reaction has to be handled immediately. You fill out a post-treatment form with all the details and give a copy to the National Drug Authority. It must be handled immediately, that we know. (DA3)
		**Respondent:** That is part of the reason, one we don't prepare them. Two, the higher the worm load, the higher the reaction and that is why I was saying we don't give feedback, we don't go to look at what outcome, we refuse the many perceptions that this drug is the one making me sick not knowing that the drug has killed many dead bodies in the stomach so their way of coming out is those proteins reacting with their bodies. Because when they die, they leave a few toxins in you.<br>**Interviewer: Because some of them think it is an overdose.**<br>**Respondent**: Yes, they think it is an overdose. They think it is the tablet which is bad, whereas it is the hormones, while we were doing onchocerciasis campaigns, we would test to see the level of hormones, so

		we would compare, this one is low, this one is high, who reacts? Of course, the one who is high reacts. So how do you counter-react what we don't give? We treat and leave them to suffer and conclude themselves but if we were treated, come back and say are you okay, you say I'm not okay, take anti-reactant, you balance.<br>**Interviewer: Why can't the government give them some drug that helps them to manage the reaction?**<br>**Respondent:** That is the antihistamine just (anti-reactants), and somebody will be happy and say when I took their drug they came back and they followed up. So we need to design a program which is exhaustive not these half ones you take someone, before he crosses the road, for you have gone back, maybe he has been knocked or he has not reached where he is going. (DA4)<br>[…]<br>**Interviewer: Is there a possible way that this praziquantel can be given with the anti-reactant?**<br>**Respondent:** It is the cost, so we have to look at case by case because if we say piriton, it is costly. (DA4)
		Let us say if we have had drugs coming, my transport is a problem. NTD, when they had just started, they gave me a motorcycle, which is now very old. So, if anything, the program could also plan for a motorcycle for my easy transport. Because if they call here, and I am talking of adverse side effects, you ride very fast to [name of bigger village], but if there is no transport, you find that you won't act very fast. (DA2)
Health system financing	Lack of funding	Then, the medicine distribution, the challenge is in terms of funding fuel. (DA4)
		We don't have the wage money, money, for recruiting health workers. The next one is we don't have equipment in the facility (DA1).
		Then, maybe the one is finance or financial support. If you don't have financial support, then these people will not be treated. Like our district is a local district, we have no funds, enough funds. They can't run such a program, which can cost a lot of money. Procuring drugs, giving people some support like the VHTs, even the technical staff to go down to health educate, then give out the drugs, etc. You may find out that at the local district, they cannot do it if you people don't come in to help (DA2).
Health workforce	Facilitation of VHTs	VHTs are unpaid volunteers; however, they receive little facilitation (~5.000 UGX) to boost their morale. VHTs argue their work is hectic and generally ask for higher payments, like 10.000 or 20.000 (DA2).<br>In the past, providing t-shirts to VHTs had an impact. They wore them like a uniform, which can help them increase their visibility and status. Providing gum boots and umbrellas would facilitate them working well during rain and sunshine (DA2).
	Unsuitability of VHTs	One of them is empowering them with knowledge, two is facilitating them nicely and that facilitation is to do with the conclusive activities that I have talked about. We have monitored, we have evaluated, what are

		the results, why don't you give us the feedback? Even VHTs, by the way, complain about how did we perform, so we just snatch our data away from them and leave them. (DA4)
		They [VHTs] will have the knowledge but will not make an impact because if you're to compare with Jesus, he could not preach in his own village. He was popular in another village, but not in his village. So, when you say my neighbour comes and teaches you, they assume you're wasting your time, but if they say the [name of health inspector] is coming at two o'clock, all the villagers become eager because they respect him. (DA4)
	VHTs training	For them [VHTs] actually this a new formulation and it's a new strategy, completely very new from what we have been doing, so they need to be trained very well, how to handle this delicate class, this subgroup of people, they need to be trained in terms of management and terms of data collection and I know the program is well prepared to look at some key issues, in terms of drug administration, having the key equipment for drug administration. (DA6)
	Training general	I will be part of the social mobilisation and sensitisation, I will be part of the advocacy team, I will be part of the training of trainers, I will do monitoring, which should give feedback meetings, that there is also a big gap when we treat, we get our data and we take, we do not go back to tell them how we performed, we don't go back to look at them, to ask them how they are feeling, that relationship also we should put it on board. (DA4)
		My involvement will be in mobilisation and health education. Training or skilling the lower cadres at the sub-county level downwards. And even support supervision during the implementation. (DA3)
		Now about those mass campaigns, first, they train the district supervisors who go and train the lower cadres at the subcounty. They are supposed to supervise the VHTs, the local leaders (LCs) because each one has roles and responsibilities to play. (DA3)
		The VHTs should be oriented on bilharzia, what is bilharzia, the causes of bilharzia, because we don't give them a lot of knowledge. Tell them what bilharzia is, the disease distribution in the community where they live, what are the causes, what are the preventive measures, and the treatment. (DA1)
		What I know, the pieces of training are sometimes inadequate depending on who's training them, because all these packages we give them in one day, whereas I'd prefer we give them the dozes and they do it practically. For example, we teach, one of the topics is the disease itself, how does it look like, how is it transmitted, how do you control it, and what is its effect. The other one we talk about is records keeping, how do you record/ register the households after registering, and how do knock out the below five years? After knocking out, how do you calculate the amount of medicine you require? After that, you're going to treat, how do you tally, how do you interpret the tally, how do you sum up the tally? You go to drugs, you

		look at what you got first, and then what you got at the end, can you correlate it to what you have treated so that's the kind of thing we do. (DA6)
	Managing side effects	Yes, when we are looking at the health workers as key stakeholders, we are looking at observing these side effects, actually filling the pharma vigilance forms, because it is a new strategy introduced, so it needs a lot of care and a lot of information to be collected in terms of reactions. (DA6)
	Partly poor supervision	One other challenge is at the office here, because there is poor supervision. It is even the cause of absenteeism. If someone knows they supervise her, she cannot risk going out of the station. So poor supervision has led to people relaxing about their responsibilities and do otherwise, because they know no one is following them up. (DA3)
Access to essential medicines	Non-availability or shortage of medicine	…actually, when drugs delay, people demand. They say, 'our drugs have delayed,' so it means it is fine. Though sometimes things [happen], which are outside our reach. For instance, we are not able to access drugs unless it is under a program. They do not come as part of our essential kits from NMS, so the presence is determined by who donates the drugs. Probably, if we are to own it as a district, then it is only having a schedule like it happens on child's days. Then we request the drugs from NMS, but you find that drugs are not there, and we need to follow the availability of the drugs to determine the existence of the program. (DA6)
		Shortage of medicine, drugs, medicines, that's another challenge. (DA1)
Health information systems	Misdiagnosis leads to an underestimation of the prevalence	For us as a health district, we offer an integrated package whereas actually, in some communities, when you offer such an integrated package, there turns out to be an interest in terms of who is giving the package. My interest may be in malaria, so when I go to give a package, I will specifically give the biggest message on malaria – and I tend to ignore bilharzia. This is evidenced in our systems; when you go into the health facilities, rarely will you find requests being made for the diagnosis of bilharzia, because this is important for any bloody diarrhoea or anything similar should immediately be related. And such requests should be very high. But when you look at the suspicion index in terms of diagnosis, the suspicion index is still very low in our facilities. That means the continuous capacity building is still very important, and of course, there should be targeted capacity building of the systems, especially the health facility system. They only receive one or two days of training during MDA, and that's all, but there should always be a

		targeted training encompassing diagnosis, encompassing history taking, just as you know malaria has gone, malaria has developed very good indicators, test and treat. [...] If we could adopt similar methodologies like every case of abdominal diarrhoea and whatever, as long as it's from the lake shores, it must be suspected for schistosomiasis and then take a test. We would be looking at indicators like how many were tested for bilharzia this month and how many turned positive. Now, the evaluations would be taking place within the system in terms of the indicators. The MDA indicators are very good, but if they turn out to be routine, then they remain for planning, looking at endemicity, looking at morbidity levels, but systemic management of schistosomiasis, I think, should be brought on board. (DA6)
	Problems with data quality and linking different registries	They do both, and usually, they start with the witch doctor, they use all the medicines they want, and it does not work, and when there are chances that they go to the facility, they will be diagnosed, and usually our facilities, I think we have a gap there with our health workers, because when they see people passing out 'whatever', passing bloody urine and bloody diarrhoea, they say Ebola and they refer. And at the end of it, all they diagnose something different, because Ebola has been there and we suffer a lot from it. They don't think very fast about bilharzia, rarely do they if you went to those facilities along there in [village name A], [village name B], [Village name with a Health Centre III] and you go to their register, you can hardly find bilharzia. (DA5)
		I think I have had an experience where I participated in the death audit at the hospital, you find a very big knowledge gap within the consultants, and this is a case which comes to the hospital, remains in the hospital with all the signs and symptoms of malaria, the consultants keeps insisting that this is severe malaria, anaemia comes in, they do malaria, malaria is not there. Then when you look, when you are analysing the history of this case, you find that this is a case which has come from the landing site, differential diagnosis never showed schistosomiasis. No request was suspected to have been bilharzia, yet actually, this was a person who is becoming anaemic due to schistosomiasis, so they continue giving anti-malarial and in the end this person dies, but when you are at an audit time, you realise, like at a program level, I know what it means, it means there is a misdiagnosis. And this is a knowledge gap, because at a regional referral, this people are referred especially those that are starting to develop, but the regional referral would go into what is called 'tapping', so there is that knowledge gap especially at that, at community VHTs are very well informed, even if they see someone swelling 'they say ah, that one bilharzia', someone vomiting blood, 'isn't that one bilharzia', but when you come at facility, you find that gap. We only need to bridge that one, and then our reporting system will start reflecting. The flag will be red, and you will see that there are a lot of bilharzia cases, but that flag you go to the institute is not there. That's one of the failures. (DA6)

		For data collection, the challenge is with entry. The moment you enter the wrong data, then that's it. So, at times when we are collecting data, you find that we gave someone ten tablets, and they're accounting for twelve. (DA4)
		Data management, health information systems, functionality, and not fully functioning are data issues. It's a challenge. (DA1)
	Lack of feedback for reported cases of pharmacovigilance/ side effects	In terms of reporting, like for example, at the community level, our VHTs are on a stand alert, they are always vibrant to report whatever they suspect as an illness. But we are being let down by our own, because the referral systems are not streamlined. They are still lacking, for example, if you refer to the health centre, what happened thereafter. Or then, if you refer to the main health referral, what happened, so somebody refers a patient and does not know what follows whether the patient went or did not go. (DA5)
Parental acceptance of the new health programme	Generally, high acceptance expected	On schistosomiasis, they have not been treating children [under five], whereas those children are also exposed. Their parents have been demanding and challenging us: “I go with my child to the water, and I go with my child to the garden. Why can’t we treat them?” So, my opinion is that they will welcome the program. It is a matter of sensitising them, telling them the right thing, and showing examples. You know in communities examples are very important, so they will take it up. (DA4)
		When drugs delay, people demand, they say ‘our drugs have delayed’ so it means it is fine. Though sometimes things [happen], which are outside our reach. For instance, we are not able to access drugs unless it is under a program, they do not come as part of our essential kits from NMS, so the presence is determined by who donates the drugs and probably if we are to own it as a district, then it is only having a schedule like it happens on child’s days. Then we request the drugs from NMS, but you find that drugs are not there and we need to follow the availability of the drugs to determine the existence of the program (DA6).<br>I believe the acceptance level will be very high. [...] I do not anticipate much except for when we introduce it, and there happen to be severe side effects, because that one can create stigma and lead to some caretakers shying away (DA6).

	Concerns due to historical incidences	Now, some parents have heard them say that because some parents are older than me, this could have happened before me. There are scenarios where they have had treatments, and there is mass death for children. And actually, there is milk that came at one point, these supermarket foods and children took that milk and got TB. But of course, this is not authentic information, because you cannot look for where it was written that it happened. But as parents, they want to keep close to their children. You must have enough explanation. That is why I said I must be very knowledgeable and even share information that it has happened through research and it has happened elsewhere, and it has worked. And actually, when I am talking to them, I say with science, there is no forgery; it is proven information. So, it is safe for the children, because nobody would want the children to die, because they are the leaders of tomorrow. (DA5)
	Concerns of overmedication	**Respondent:** You see, children have been given a lot of types of drugs.<br>**Interviewer: Of bilharzia?**<br>**Respondent**: No, I am talking in terms of all types of treatment. There is that of immunization. At first, they were only giving for 6 immunisable diseases, but now we are preventing twelve, all those are drugs given into one body of a child. There is treatment for other diseases, so there is a need for a lot of convincing. We need a lot of sensitisation, because they have seen a lot, and one may say: "You have given a lot of medicine to children, measles, TB, Polio, etc. This is too much for the children!" They will have to say that. So, there is a need to reassure them and convince them, that this is a different drug. (DA3)
	Association of bilharzia with witchcraft	**Interviewer: So, would they then go to the health facility, or would they go and do traditional things?**<br>**Respondent:** They do both, and usually, they start with the witch doctor, they use all the medicines they want, and it does not work, and when there are chances that they go to the facility, they will be diagnosed. And usually our facilities, I think we have a gap there with our health workers, because when they see people passing out 'whatever', passing bloody urine and bloody diarrhoea, they say Ebola and they refer and at the end of it all they diagnose something different because Ebola has been there and we suffer a lot from it. They don't think very fast about bilharzia, rarely do they if you went to those facilities along there in [village A], [village B], [village C] and you go to their register, you can hardly find bilharzia.
	Political disparities	Then, when we are going to do that, we have to look at who can help us to do this, the political will, because the politicians can spoil everything (DA5).
		We also have a problem with politicians; that's now politics. You may find that somebody may not support a program because the government that's in power is against it. So you find that whatever program comes, he may tend to shy away from that program and won't support it. (DA2)
	Hesitant groups	Then, we also have a challenge regarding religion. Religious sects. We have a sect called Njiri Nkaru (dry gospel). These people don't believe in treatment. They don't want to hear or support any government

		program. They don't want to say, even swallowing tablets, this sect, they are already protected by the powers of the lord. (DA2)
		Of course, we deal with migrant communities sometimes, and they are suspicious of the new interventions, they think it's a way to actually harm them, and especially right now, there are a lot of Congolese at the lake shores who come across. And one thing I also anticipate, the community may not be all enthusiastic at once, as there are those who would want to observe what would happen to those who have received. So in the first instance, the average may not be very good, because there are those who would want to know what would happen to those who have taken the drug. It's like the way we have been experiencing the covid vaccination. They later see how the first batch would react, then we shall follow. So am anticipating that people acceptance level may not happen at the same time, but it will grow gradually by observing what happens with those who have taken the drug (DA6).<br>[...]<br>The graph has changed, because if the community can demand the drug, then such misconceptions remain at an individual level, except in some communities, where we have constant immigration, people come in from Congo. And those ones who are not informed, they tend to have different beliefs that they have been bewitched, but this one, I think one of the challenges is that as much we have health promotion, there is no continuous health promotion messages targeting specifically bilharzia. (DA6)
		The issues to deal with information and especially information that is written, like placards, where there are messages. I did not see that come out properly for the general public, I did not see a good IEC [information, education, and communication material] being given out. (DA5)
	Being busy with other activities	They don't appreciate the program, the drug distribution program. They are also busy with other things related to income and so on; you might find that they miss out on such programmes. (DA1)
	Fear of side effects	**Respondent:** Maybe side effects, a few get side effects. I had a child, I think, who almost died because of side effects.<br>**Interviewer: And how do the people react to this? How do they perceive it when they get side effects?**<br>**Respondent:** They only think the drug is bad because of the bad side effect, it has caused. So, that's what we are going through at the moment. (DA1)
	Recommended measures	Actually, there need to be inception meetings at various levels, because this is very new. It can be at district levels, then cascaded to other levels and including the community, and then we could probably also be informed through feedback from community dialogues. This is what we are introducing. What are the people saying? (DA6)

		I put up my hand and said, 'The main stakeholder is the parent, and the key stakeholder must understand the principle from the beginning – then they accept'. So, before we do this, our parents should be educated. (DA5)
		Go ahead to develop drama plays with all sessions talking about bilharzia.<br>Make clear messages that bilharzia is not out of witchcraft but a medical problem/disease.<br>We should have posters on bilharzia in schools and religious institutions to enable those who can read to get messages. Show pictures of children and adults who had bilharzia, got treatment and got better. (DA3)
		Tell the parents we train them, make them aware of the radios and produce information, education and communication materials. We can also have things like health talks; they are very expensive, but you can have them in these isolated places where the problem is. So, awareness creation and knowledge building among the population are very, very important. (DA1)
